# Round the Hospitals

**Published:** 1918-02-16

**Authors:** 


### THE MATRONS' AND SISTERS' DEPARTMENT.

### ROUND THE HOSPITALS.

The meeting at the West End Cinema, Coventry Street, W., on Friday, the 8th instant, at which the Duchess of Bedford presided, was full of interest. It revealed some surprises, and two new films, that of the Admiralty and the Air Service it controls being probably the best thing of the kind yet produced. The Duchess of Bedford made an excellent chairman, and spoke with terseness and conviction. Her Grace is evidently a believer in the College of Nursing and its importance to the nation, and to-day, is there any independent person who does not share her Grace's views? The progress made by the College in the last six weeks is remarkable, and points to an early and complete success. Those responsible for the organisation of this meeting deserve warm congratulations. It forecasts a continuous and increasing success for similar meetings on behalf of the College of Nursing. Mr. Pett Ridge had the happy thought of raising £500 in five minutes, by gifts of £100 from the audience, and succeeded before four minutes were out—a remarkable success. The further fact that £1,400 was handed in in large sums, apart altogether from smaller gifts, in addition to the proceeds of the performances throughout the day, indicates a most excellent send-off for the College meetings which are yet to come. Mr. Ben Tillett, M.P., took a strong line and upheld the rights and excellences of British nurses, who, he claimed, were entitled to the fullest recognition, and the most generous appreciation by all classes of the people and the State.

---

A new and attractive speaker at Friday's meeting was Miss E. Musson, R.R.C., matron of the Birmingham General Hospital. She gave an interesting *résumé* of the origin and present position of trained nursing, and spoke forcibly and convincingly of the claims of a probationer, and her right to obtain through every hospital board systematic training under qualified teachers, authorised examinations, and the generous treatment which was her due. The College of Nursing had special value, in its power to support probationers and all nurses who joined as members. It would enforce a uniform curriculum of training, so that badly managed hospitals must become efficient, or fail to obtain probationers and to exist as hospitals in the best meaning of the word. She made a good point against the "axes to grind" party who had sneered at the British Women's Hospital Committee because many of its members belong to the theatrical profession. Miss Musson assured them and the audience, however, that no one loved a visit to the theatre more than the best of nurses did, and they constituted an audience which had a high respect for actors and actresses, to whom they were indebted for invaluable relaxation and genuine enjoyment. Miss Compton's speech was a feature of the meeting. It was bright, forcible, and contained a few excellent stories to the point. She claimed that there was no charity whatever in contributing to the Nation's Fund for Nurses, because everybody owed a debt to British nurses, and that debt must and would be joyfully and generously paid through the British Women's Hospital Committee.

---

In this connection we may mention that the Liverpool Centre of the College of Nursing will have a field-day at the Town Hall, Liverpool, on Friday, February 22, at 2.30 p.m., when the Right Hon. the Lord Mayor will preside. Amongst the speakers will be the Hon. Sir Arthur Stanley, G.B.E., M.P.; the Viscountess Cowdray, honorary treasurer of the Nation's Fund; Miss Cummins, matron of the Royal Infirmary, who is a member of the Council of the College of Nursing; and Miss Alison Garland, who is a most active supporter of the College of Nursing.

---

The emoluments of sisters and nurses in London, with which we dealt in our issues of January 19, page 338, and January 26, page 356, have excited very great interest. In dealing with St. Bartholomew's Hospital we stated, "The sisters start at £65, rising by £6 10s. after every completed five years' service to £84 10s. The out-patient sister starts at £71 10s., rising by the same increments to £84 10s." This statement was not complete, for the reason (1) that the actual payments and allowances up to 1916 were as follows:—

The sisters were paid:—

| | |
|---|---|
| Commencing at 25s. per week:— | With dinner, uniform, and washing. |
| After 5 years' service 27s. 6d. per week | |
| " 10 " " 30s. " " | |
| " 15 " " 32s. 6d. " " | |

(2) That in December 1916 a war bonus of 3s. per week was granted to each sister; and (3) that subsequently, in consequence of the difficulty experienced in obtaining many articles of food, this bonus has been discontinued and a certain allowance of provisions has now been made to them, as follows: Per week: 3 rashers of bacon (weight approximately 6 oz.); 3 eggs; 1 sausage; ¼ lb. margarine; ¼ lb. sugar; 3 oz. tea; and 3½ pints milk. The importance of this information must be apparent, as the statement in its original form in regard to St. Bartholomew's Hospital sisters, as published in the paragraph on page 338 of The Hospital of January 19, was incomplete, and was consequently, for comparative purposes, misleading. Now our readers have had all the facts relating to sisters' and nurses' emoluments at St. Bartholomew's Hospital, St. Thomas's Hospital (January 19, page 338), and the London Hospital respectively (January 19, page 338, and January 26, page 356). It is essential that the emoluments of sisters and nurses everywhere throughout the country should be revised, and we shall be glad to have full particulars of those hospitals where the committees have recognised the position and put their sisters' and nurses' emoluments upon a new, adequate, and up-to-date basis.

Admirable work at very small cost is being done by the Travelling Expenses Fund for the relations of the sick and wounded in the 1st Northern Hospital, Newcastle-upon-Tyne. Quite early in the war it was found that many of the wounded were deprived of the pleasure of a visit from their near relatives on account of the expenses involved in the long journey and the cost of board and lodging after arrival. The organisers of the fund work in close connection with the matron and ward sisters, who advise where help is required. Regular lodgings have been engaged where at small cost the visitors are kindly welcomed, and since the beginning of the war over 1,000 persons have in this way been enabled to travel from all parts of England, from Scotland, Ireland, and the Channel Islands, to visit their sick and wounded relations. Matrons of military hospitals are often put to extraordinary straits in the effort to arrange for visits from patients' friends, and this is work in which outside folk might well follow the lead of Newcastle and take a sympathetic part.

